# 

**Published:** 1889-05

**Authors:** 


WHOLE NUMBER, CCCXXXIV.

New Series.

May, 1889.

VOL. XXVIII. No. 10.

LIBRARY
INDEXED
S.G.O.

### BUFFALO MEDICAL AND SURGICAL JOURNAL.

Established 1845 by AUSTIN FLINT, M. D.

Editors:

THOS. LOTHROP, M. D. W. W. POTTER, M. D.

Associate Editors:

FLOYD S. CREGO, M. D. EDWARD B. ANGELL, M. D., ROCHESTER, N.Y.

PUBLISHED MONTHLY.

SCRIPTA FERUNT ANNOS

BUFFALO:

1889.

Entered at the Post-Office at Buffalo, N. Y., as Second-class Mail Matter.

*Times Printing House, Buffalo, N. Y.*

